# Acute Pancreatitis: A Rare Complication of Colonoscopy

**DOI:** 10.7759/cureus.22128

**Published:** 2022-02-11

**Authors:** Saima H Shawl, Usama Bilal, Chandra Essar Mal, Veera Durga Vaishnavi Kurra, Romil Singh

**Affiliations:** 1 Internal Medicine, Chittagong Medical College, Chittagong, BGD; 2 Internal Medicine, Shaikh Khalifa bin Zayed Al-Nahyan Medical College, Lahore, PAK; 3 Internal Medicine, Liaquat University of Medical and Health Sciences, Jamshoro, PAK; 4 Internal Medicine, All India Institute of Medical Sciences, Bhopal, IND; 5 Critical Care, Allegheny Health Network, Pittsburgh, USA

**Keywords:** endoscopy, pancreatitis, post-colonoscopy, colonoscopy complications, acute pancreatitis

## Abstract

Colonoscopy is a well-tolerated therapeutic and diagnostic procedure. Although colonoscopy is relatively safe, a few complications have been reported. Abdominal pain after colonoscopy is one of the most reported symptoms, and acute pancreatitis is uncommon after colonoscopy. We present a case of acute pancreatitis in a 51-year-old female who presented with a complaint of melena. She underwent colonoscopy to rule out lower gastrointestinal pathology and developed sudden onset diffuse abdominal pain and vomiting two hours after the procedure. She was diagnosed with colonoscopy-induced acute pancreatitis based on physical examination and detailed investigations after ruling out all other potential causes. She was treated conservatively with bowel rest, intravenous fluids, analgesic, and prophylactic antibiotics. Abdominal symptoms improved quickly in a few days with complete resolution of abdominal pain, fever, and normalization of serum amylase and lipase. Early recognition and diagnosis can lead to successful treatment, and the patients should be informed about the possibility of this complication before undergoing colonoscopy.

## Introduction

Colonoscopy is a relatively common diagnostic and therapeutic procedure to investigate colonic pathology. While colonoscopy is generally considered a safe, effective, and well-tolerated procedure, it has also been associated with a few complications [1]. Common side effects include transient gastrointestinal manifestations, including nausea, abdominal pain, diarrhea, and adverse events related to anesthesia and analgesia reported by 33% of the patients [1,2]. Major complications include appendicitis, diverticulitis, bowel perforation, infection, splenic rupture, bleeding, and post-polypectomy syndrome [2]. Acute pancreatitis is one of the most common documented complications associated with upper gastrointestinal tract endoscopic procedures [3]. However, acute pancreatitis after colonoscopy is not widely reported in the literature, and only a few cases have been underlined regarding the development of post-colonoscopy acute pancreatitis [4-7]. Herein, we describe a rare case of acute pancreatitis following colonoscopy.

## Case presentation

A 51-year-old female with a past medical history of diabetes mellitus and hypertension was admitted to the hospital for the evaluation of dark color stool. She was compliant with insulin and lisinopril, and she denied alcohol use, smoking, and illicit drug abuse and reported no history of trauma. On examination, the abdominal examination was non-significant, and coffee-ground appearance feces with positive occult blood test were noted in the rectal vault by digital rectal examination. Her serum chemistry and blood results were normal except for low hemoglobin (9.8 mg/dL). She was found to have ulcers in the stomach and treated appropriately. Later on, she was scheduled for a colonoscopy due to her father's family history of colon cancer and universal screening. Her bowel was prepared using polyethylene glycol in 2 L of water. As premedication, midazolam and propofol with a single bolus of 50 mg were given over 10 seconds through a rapid running intravenous catheter. Colonoscopy was guided up to the terminal ileum, and there was no difficulty crossing the ileocecal valve, and the total intubation time was 10 minutes. Findings revealed one sessile polyp in the sigmoid colon and one polyp in the ascending colon. Both polyps were removed using a cold snare, and electrocautery was not performed during the procedure.

The patient developed sudden onset diffuse abdominal two hours after the procedure. Abdominal pain was sharp, 8/10 in severity, radiating to the back followed by nausea, and two episodes of vomiting, containing a clear liquid. This was her first time to have such pain in her life. She was febrile with a heart rate of 98/minute, respiratory rate of 25/minute and blood pressure of 130/90 mmHg. On examination, she had epigastric tenderness with no signs of organomegaly and peritonitis. Cardiovascular and respiratory examinations were unremarkable. The result of blood tests showed an elevated leukocyte count (12,100 cells/mm^3^), lipase (1619 IU/L), amylase (502 IU/L), and C-reactive protein (31 mg/L) (Table 1). The lipid profile, serum calcium, and parathyroid hormone levels were normal (Table 2).

**Table 1:** Laboratory results before and after colonoscopy

Parameter	Before colonoscopy	After colonoscopy	Reference value
White cell count	6900	12,100	4000-11,000 cells/mm^3^
Red cell count	4.9	4.8	4.45-5.65 million cells/mm^3^
Lipase	140	1619	0-160 IU/L
Amylase	88	502	30-110 IU/L
C-reactive protein	09	31	0-5 mg/L
Alanine aminotransferase	37	39	7-55 IU/L
Aspartate aminotransferase	30	32	8-48 IU/L
Alkaline phosphatase	68	71	36-92 mg/dL
Prothrombin time	11.5	11.4	11-13.5 seconds
Partial thromboplastin time	32	35	30-40 seconds
Total bilirubin	1.2	1.4	0.3-1.3 mg/dL
Serum creatinine	0.8	1.1	0.7-1.2 mg/dL
Blood urea nitrogen	15	17	14-20 mg/dL

**Table 2:** Results of lipid profile and serum calcium and parathyroid hormone levels LDL: low-density lipoprotein, HDL: high-density lipoprotein

Parameter	Lab value	Reference range
Serum calcium	9.2	9.0-10.5 mg/dL
LDL	59	<130 mg/dL
HDL	59	>55 mg/dL
Serum triglyceride	147	<150 mg/dL
Serum cholesterol	202	<200 mg/dL
Parathyroid hormone	29	14-65 pg/ml

**Figure 1:**
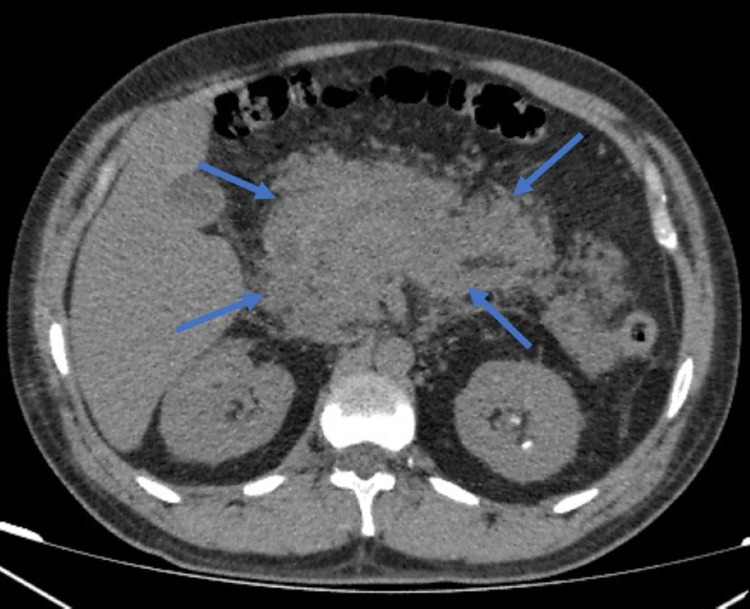
Abdomen CT demonstrating diffuse enlargement of pancreas with ill-defined borders (arrows)

An erect abdominal x-ray was performed, which revealed no free air under the diaphragm. The pancreas was not visualized on abdominal ultrasound, liver and biliary ducts abnormalities were not reported, and gallstones and biliary stones were not found. Computed tomography (CT) of the abdomen was performed, which showed edematous pancreas and poorly defined borders consistent with acute pancreatitis diagnosis (Figure 1). There were no local complications or organ dysfunctions, and the CT severity index (CTSI) score was 6. The patient was kept nothing by mouth and was treated conservatively with bowel rest, intravenous fluids, analgesic, antiemetic, and prophylactic antibiotics; improvement in her condition was noted. Lipase and amylase levels were monitored daily. Abdominal symptoms improved quickly in a few days with complete resolution of abdominal pain, fever, and normalization of serum amylase and lipase levels in seven days. She was discharged one week later with a follow-up.

## Discussion

Acute pancreatitis is an inflammation of the pancreas characterized by epigastric pain, nausea, vomiting, and elevated serum amylase and lipase levels on the complete metabolic panel [8]. The incidence of acute pancreatitis is rapidly rising due to obesity and gallstone and ranges from 5 to 80 per 100,000 population, with the highest incidence seen in the United States [9]. There are many etiologic factors for acute pancreatitis in the general population, including alcohol abuse, gallstones, metabolic disorders, autoimmune disorders, medication side effects, surgery, trauma, and infections [10]. Acute pancreatitis is also documented after endoscopic procedures related to upper gastrointestinal pathology, and low-grade pancreatic inflammation following endoscopy may be more prevalent than previously believed [11]. In previous studies, patients undergoing colonoscopy showed asymptomatic elevated amylase levels in the urine of 6.6% of patients and blood of 12% of patients [11,12]. However, none of these patients reported symptoms of acute pancreatitis. Acute pancreatitis after colonoscopy is uncommon, and a literature review highlighted only four cases related to acute pancreatitis following colonoscopy [4-7]. Colonoscopy was particularly challenging in one case, and the endoscopist encountered difficulties passing the scope through the splenic flexure, which required multiple attempts [4]. While in another case report, acute pancreatitis was thought to be caused by indirect trauma due to hemorrhage around the pancreatic tail in CT imaging [5]. Another case report showed that a patient with a history of inflammatory bowel disease, recurrent acute pancreatitis, and use of immunosuppressants experienced pancreatitis after colonoscopy [7]. These factors suggested that the disease might have multifactorial etiology.

The pathophysiology for the development of acute pancreatitis in such cases is uncertain, and it has been proposed that moving the endoscope through the bowel causes indirect injury to the body and tail of the pancreas due to the anatomic proximity of splenic flexure to the pancreatic tail [5,13]. Pancreatic injury may occur due to excessive bowel distension caused by gas insufflation. Furthermore, excessive external pressure may also trigger local trauma and inflammatory response. A second explanation could be the use of electrocautery during polypectomy, which is capable of causing transmural burns, mechanical trauma, and irritation to the pancreas and may precipitate an inflammatory response resulting in acute pancreatitis [11].

In this case, the patient developed acute pancreatitis directly after a colonoscopy. She was healthy and fit previously. The procedure went smoothly, with easy passage through the colon without electrocautery and external abdominal pressure. She had no other risk factors for acute pancreatitis, and her lipid profile, autoimmune screening, and metabolic panel were non-significant. Before the procedure, she did not report any abdominal trauma, and her imaging revealed no pancreatic anatomic anomalies. It is unlikely that the patient had propofol-induced acute pancreatitis because our patient received a single dose of 50 mg propofol through rapid infusion. The literature reported that a minimum dose of 200 mg with intermediate latency of 1-30 days could lead to acute pancreatitis [14]. It is also implausible that statin was the etiology of acute pancreatitis, as she had been on atorvastatin for more than four years; statin-induced acute pancreatitis is reported mostly within months of commencing the therapy, and her condition did not worsen after rechallenging again with atorvastatin therapy [15].

## Conclusions

Abdominal pain is common after colonoscopy and warrants evaluation to rule out causes such as perforation. Although acute pancreatitis induced by colonoscopy is rare, it should be considered among the differential diagnoses after ruling out all probable causes. Early recognition and diagnosis can lead to successful treatment, and the patients should be informed about the possibility of this complication before undergoing colonoscopy.

## References

[1] Ko CW, Dominitz JA (2010). Complications of colonoscopy: magnitude and management. Gastrointest Endosc Clin N Am.

[2] Reumkens A, Rondagh EJ, Bakker CM, Winkens B, Masclee AA, Sanduleanu S (2016). Post-colonoscopy complications: a systematic review, time trends, and meta-analysis of population-based studies. Am J Gastroenterol.

[3] Pekgöz M (2019). Post-endoscopic retrograde cholangiopancreatography pancreatitis: a systematic review for prevention and treatment. World J Gastroenterol.

[4] Ko HH, Jamieson T, Bressler B (2009). Acute pancreatitis and ileus post colonoscopy. Can J Gastroenterol.

[5] Nair SP, Debnath P, Udgirkar S (2020). Acute pancreatitis: a rare post-colonoscopy sequela. Clin Endosc.

[6] Sidiqi MM, Gong B (2019). Acute pancreatitis as a complication of routine colonoscopy—a rare case report. Int J Surg Case Rep.

[7] Limb C, Ibrahim IA, Fitzsimmons S, Harper AJ (2016). Recurrent pancreatitis after unremarkable colonoscopy, temporalised by CT imaging: an unusual case. BMJ Case Rep.

[8] Kataria S, Sharif A, Ur Rehman A, Ahmed Z, Hanan A (2020). COVID-19 induced acute pancreatitis: a case report and literature review. Cureus.

[9] Ouyang G, Pan G, Liu Q (2020). The global, regional, and national burden of pancreatitis in 195 countries and territories, 1990-2017: a systematic analysis for the Global Burden of Disease Study 2017. BMC Med.

[10] Chatila AT, Bilal M, Guturu P (2019). Evaluation and management of acute pancreatitis. World J Clin Cases.

[11] Nevins AB, Keeffe EB (2002). Acute pancreatitis after gastrointestinal endoscopy. J Clin Gastroenterol.

[12] Kobayashi T, Fukuchi S, Sawano S, Yamada N, Ikenaga T, Sugimoto T (1979). Changes in serum isoamylase activities after fibergastroduodenoscopy and colonoscopy: isoamylase after FGDS and FCS. Endoscopy.

[13] Honda K, Mizutani T, Nakamura K (2006). Acute pancreatitis associated with peroral double-balloon enteroscopy: a case report. World J Gastroenterol.

[14] Haffar S, Kaur RJ, Garg SK, Hyder JA, Murad MH, Abu Dayyeh BK, Bazerbachi F (2019). Acute pancreatitis associated with intravenous administration of propofol: evaluation of causality in a systematic review of the literature. Gastroenterol Rep (Oxf).

[15] Singh S, Loke YK (2006). Statins and pancreatitis: a systematic review of observational studies and spontaneous case reports. Drug Saf.

